# Walking-induced inertial effects on the cardiovascular system

**DOI:** 10.3389/fnetp.2025.1637551

**Published:** 2025-09-25

**Authors:** Aurora Rosato, Emanuele Perra, Eric Rullman, Seraina A. Dual

**Affiliations:** ^1^ Intelligent Heart Technology Lab, Department of Biomedical Engineering and Health Systems, KTH Royal Institute of Technology, Stockholm, Sweden; ^2^ Division of Clinical Physiology, Department of Laboratory Medicine, Karolinska Institute, Stockholm, Sweden

**Keywords:** lumped parameter modeling, cardiovascular modeling, walking, baroreflex, physiological network, cardiac locomotor coupling, network physiology, hemodynamic

## Abstract

**Introduction:**

During exercise, the cardiovascular, respiratory, and locomotor systems interplay dynamically, yet the specific mechanisms of cardiovascular and locomotor interaction during simple rhythmic exercise like walking remain unclear. Computational models constitute a powerful tool to investigate the interplay of networked physiological systems, but while gravitational and postural effects on circulation have been explored, the influence of inertial forces from body motion on hemodynamics has not been addressed.

**Methods:**

Here, we present a closed-loop cardiovascular model that incorporates inertial effects during walking. The lumped parameter model includes 25 vascular compartments, a four-chamber heart with valves, pericardial and intrathoracic pressures, interventricular septal dynamics, and a baroreflex mechanism. Inertial effects are modeled as additional hydrodynamic pressure sources in each vascular segment, equivalent to the acceleration of blood mass, caused by gravity and motion. Three protocols are used: a head-up tilt test to validate baroreflex and gravity effects; a synthetic walking simulation with controlled heart rate (HR) and step rate (SR); and a human walking experiment (n=2) linking beat-wise simulated aortic pressure to measured brachial pressure using recorded HR and body acceleration. Beat-wise morphology similarity (K-stat) between experimental and simulated hemodynamic waveforms is quantified with a two-sample Kolmogorov-Smirnov test.

**Results:**

The model reproduces expected physiological responses to head-up tilt. During synthetic walking, inertial effects result in pressure augmentation, increasing systolic or diastolic pressure depending on the phase between HR and SR. With SR > HR, phase variability produces a low-frequency “beating” in the pressure waveforms and mean arterial pressure, corresponding to the difference between SR and HR. In the human subject experiment, the model accurately replicates beat-wise pressure changes at varying phase shifts between HR and SR. Quantitative comparison shows a substantial increase in similarity of waveform when hydrodynamic pressure is included (K-stat: 0.123 vs. 0.029 for P1; 0.164 vs. 0.059 for P2).

**Conclusion:**

Introducing contributions of body acceleration as an additional hydrodynamic pressure source in the vascular compartments seems a valid way to capture walking-induced inertial effects. This work contributes to the broader effort to characterize physiological network adaptations to exercise and offers a foundation for future research studying and optimizing cardiac-locomotor interaction.

## 1 Introduction

Exercise functions as a perturbation that challenges the body’s interconnected physiological systems, especially the cardiovascular, respiratory, musculoskeletal, and neural. Analyzing exercise such as walking provides insights into how adaptive responses, including fatigue, recovery, and performance, emerge from systems interactions rather than isolated organ behavior (Balagué et al., 2020). Network physiology represents a recent framework utilizing statistical modeling of topological networks to characterize nonlinear feedback mechanisms and their resulting complex transient dynamics (Ivanov, 2021; Bashan et al., 2012). Complementary is the study of the biomechanical relationships of interacting physiological systems.

During rhythmic exercise such as locomotion, there is a functional interplay between the cardiovascular, respiratory and musculoskeletal systems. Recently, the study of the interaction of cardiovascular and respiratory system has seen a lot of scientific progress Fisher et al. (2022), specifically in the context of exercise and cardiorespiratory coordination (Garcia-Retortillo et al., 2019a; Garcia-Retortillo et al., 2019b). In parallel, also our understanding of the interaction between the locomotor and cardiovascular systems has advanced significantly. Recent evidence uncovers how autonomic regulation and cardiac function synchronize with muscle activation during exercise (Garcia-Retortillo and Ch Ivanov, 2024), and that beneficial metabolic effects occur when precise coordination between walking and heart relaxation is achieved (Constantini et al., 2018; Wakeham et al., 2023; Niizeki et al., 1993). Despite these advancements, the biomechanical effects resulting from the coupling between the locomotor and cardiovascular systems (cardiac-locomotor coupling) remain poorly understood. It remains unclear to what extent cardiac locomotor coupling modulates hemodynamics via inertial effects (O’Rourke and Avolio, 1992), venous muscle pumping (Zhang, 2002), and ejection of blood from intramuscular arteries during muscle activation (Walløe and Wesche, 1988; Niizeki, 2005). In turn, these hemodynamic effects drive alterations in cardiac afterload and preload (Langan, 2025; Wakeham et al., 2023), and coronary perfusion (mechanoenergetics) (Malliaras et al., 2014) as well as potentially a complex cascade of autoregulatory feedback mechanisms (Garcia-Retortillo and Ch Ivanov, 2024).

This lack of understanding currently limits our ability to propose health-effective physical exercise and rehabilitation strategies, but also poses challenges in designing cardiac support devices which work safely during daily activities.

Computational models of physiological networks are uniquely suited to explore underlying relationships. They have become a useful tool for the design and early feasibility assessment of cardiovascular devices, offering predictive insights that can reduce the extensive physical prototyping and *in vivo* studies. In clinical practice, such models are being used to assist decision-making processes and predict patient outcomes in response to therapeutic or interventional treatments (Fumagalli et al., 2024). However, their application in scenarios involving physical activity, particularly to inform cardiovascular rehabilitation strategies, remains limited. Addressing this gap, recent studies have developed models that simulate dynamic adaptation to exercise by incorporating cardio-respiratory and metabolic control mechanisms dependent on total workload (Fresiello et al., 2016). Other studies have also investigated the autoregulatory effects under gravitational stress, opening the door to studying the dynamic inertial effects of bodily acceleration during walking.

Among the first models to account for gravitational effects in cardiovascular simulations is Heldt (2004), who implemented a 0D lumped parameter multi-compartmental model in conjunction with baroreflex control dynamics to reproduce transient and steady hemodynamic responses to changes in head-up tilt angle. Similarly, Peterson et al. (2002) employed a 0D model of the cardiovascular system to quantify the influence of intrathoracic and hydrostatic pressure contributions on cardiac function across different body postures and gravitational levels, ranging from 0 to 1.8 G. More recent work has combined a 1D (Fois et al., 2022; Zhang et al., 2017) or 3D (Lau and Figueroa, 2015) arterial tree with a 0D systemic network to capture hemodynamic changes during passive head-up tilt, validating the simulation results against experimental data (Fois et al., 2022). Although gravity-induced hydrostatic effects are incorporated in many models (Diaz Artiles et al., 2016; Lau and Figueroa, 2015; Gallo et al., 2020; Fitzjerrell et al., 1983); Rafik (2021), little research addresses the inertial forces arising from dynamic body motion (Belardinelli et al., 1989; O’Rourke and Avolio, 1992) in closed-loop 0D cardiovascular models, leaving walking-induced hemodynamics insufficiently characterized.

In walking, the cardiac and locomotor systems interact in several distinct ways. During locomotion, cardiac activity supplies oxygenated blood to the working muscles, while the skeletal muscle pump, particularly the calf, enhances venous return via intermittent increases in intramuscular pressure, causing blood ejection from intramuscular veins and venous sinuses (Tauraginskii et al., 2023). Moreover, locomotion induces cyclical acceleration and deceleration of the body’s center of mass, generating inertial forces that dynamically redistribute blood volume. The body vertical motion gives rise to positive and negative pressure waves in the aorta (Nichols et al., 2022), that can constructively or destructively interfere with existing blood pressure patterns.

To date, three studies attempted to model the hemodynamics impact of accessory wave generated at each foot strike. In the first study from Palatini et al. (1989b), a chest-mounted saline-filled container was used to externally capture inertial pressure changes during running. These pressure fluctuations were synchronized and later integrated with intra-arterial pressure recordings obtained during cycling. The resulting pressure tracing closely resembled the pattern observed during overground running, showing that running gives origin to a wave of varying amplitude, whose frequency is related to the runner’s pace. In a successive study, O’Rourke and Avolio (1992) used a single tube model of uniform stiffness representing the aortic trunk and lower limb arteries. Two sinusoidal pumps were incorporated into the model to represent left ventricular ejection and leg muscle contraction and they interacted linearly at varying frequencies. The results showed that when the two pumps were entrained at the same frequency, the resulting sinusoidal waveform exhibited a peak-to-peak pressure amplitude ranging from 140 mmHg when the waves were in phase to 20 mmHg when they were 180° out of phase. Additionally, a beating phenomenon was observed when the two pumps operated at different frequencies. Finally, a model of the cardiovascular system during running has been developed to study the optimal interaction between heart contraction and muscle activity by Zhang (2002). Two key physiological mechanisms were integrated into the model: modulation of peripheral resistance due to transient blood flow obstruction during muscle contraction, and modulation of venous unstressed volume to simulate the impact of the feet on the ground. The study was able to show that stroke volume (SV) oscillates when step rate (SR) differs from heart rate (HR), and become stable when SR equal HR. Highest SV and consequent optimal interaction, was obtained when stepping happens during filling phase of the cardiac cycle. Experimental data, also revailed that under constant cardiac output (CO) conditions (same workload and oxygen consumption), HR varied with the phase, with minimal HR, and consequent higher SV, when the steps aligned with the heart’s filling phase.

In this study, we present a closed-loop cardiovascular model featuring inertial effects of body acceleration during walking. Utilizing this model, we explore how the cardiovascular and the locomotor system interplay at high similarity of HR and SR. Heart-paced walking is realistic and conveniently allows for a very controlled study of hemodynamic effects on the cardiovascular system and exhibits some interesting frequency phenomena. Finally, we validate the aortic pressure waveform including inertial effects of walking with experimentally measured hemodynamics. Importantly, the implementation of gravity is validated against state-of-the-art models during a head up tilt.

## 2 Methods

### 2.1 Closed loop model of the cardiovascular system

**FIGURE 1:**
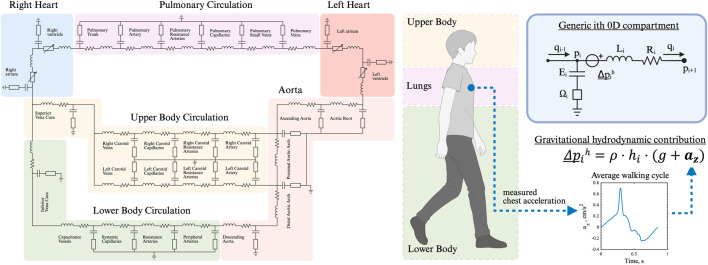
Closed-loop, multi-compartmental lumped parameter model of the cardiovascular system. Simulation of right and left heart, as well as pulmonary, upper and lower body circulation. The chest acceleration is modeled as an additional pressure source in all vertically orientated vessels.

A closed-loop lumped parameter model was developed to simulate the cardiovascular system dynamics, based on the model by Broomé et al. (2013) (Figure 1). The model consists of 25 vascular compartments representing major segments of the systemic and pulmonary circulations. Each segment is described using a four-element Windkessel model, incorporating resistance, compliance, and inertance to capture hemodynamic behavior. All Windkessel parameters are derived based on first principles from geometric vascular parameters, such as length and radius, representative of vessels in each compartment.

The heart is represented as a four-chamber pump, with active and passive myocardial mechanics modeled using a periodic double-Hill function (Stergiopulos et al., 1996). Cardiac valves are implemented with a combination of Bernoulli resistance and inertial effects, allowing smooth transitions in the valve area from open to closed states in response to transvalvular pressure gradients (Mynard et al., 2012). The model takes into account additional mechanisms such as pericardial and intrathoracic pressures (Sun et al., 1997), and interventricular septal interaction (Maughan et al., 1987).

All parameters used in the model for the heart chamber, cardiac valve, and cardiovascular compartments (resistance, compliance) are chosen based on physiological values from Broomé et al. (2013).

The model was implemented in MATLAB Simulink (MathWorks Inc., Natick, MA, USA) and numerically integrated using a fourth-order Runge–Kutta solver at a sampling frequency of 2 kHz.

#### 2.1.1 Time-varying elastance function

The time-varying elastance of each cardiac chamber, e(t) (Equation 1), is described using a double-Hill function (Stergiopulos et al., 1996; Mynard et al., 2012; Broomé et al., 2013) that captures both the contractile and passive mechanical properties of the myocardium:

$$e\left(t\right)=k\left(\frac{g_{1}}{1+g_{1}}\right)\left(\frac{1}{1+g_{2}}\right)+e_{\min}\left(v\right),$$ (1)

where

$$g_{1}={\left(\frac{t}{\alpha_{1}T}\right)}^{m_{1}},\;g_{2}={\left(\frac{t}{\alpha_{2}T}\right)}^{m_{2}}.$$ (2)

In Equation 2, T denotes the duration of the cardiac cycle. The dimensionless parameters $\alpha_{1}$ , $m_{1}$ , $\alpha_{2}$ , and $m_{2}$ define the shape of the elastance curve during the contraction and relaxation phases, respectively. The scaling factor k is chosen such that the maximum elastance satisfies $\max\left(e\left(t\right)\right)=E_{\max}$ :

$$k=\frac{e_{\max}\left(v_{ed},q\right)-e_{\min}\left(v\right)}{\max\left[\left(\frac{g_{1}}{1+g_{1}}\right)\left(\frac{1}{1+g_{2}}\right)\right]}.$$ (3)

In Equation 3, the maximum elastance, $e_{\max}$ , is modulated by the end-diastolic volume $v_{ed}$ and chamber-specific output flow q , thereby incorporating the Frank–Starling mechanism (Sun et al., 1995) as shown in Equation 4:

$$e_{\max}\left(v_{ed},q\right)=E_{\max}\cdot \left[1-{\left(\frac{v_{ed}}{V_{ed,\max}}\right)}^{4}\right]\cdot \left[1-\frac{q}{Q_{\max}}\right].$$ (4)

The minimum elastance, $e_{\min}\left(v\right)$ (Equation 5), describes the passive exponential pressure-volume relationship during diastolic filling (Chung et al., 1997):

$$e_{\min}\left(v\right)=E_{\min}\cdot e^{\sigma \cdot \left(v-v_{0}\right)}.$$ (5)

Parameters related to the cardiac function are reported for each chamber in Table 1.

**TABLE 1:** Parameters for the cardiac chambers model.

Chamber	$E_{\text{max}}$ $\left(\frac{mmHg}{mL}\right)$	$E_{\text{min}}$ $\left(\frac{mmHg}{mL}\right)$	$\alpha_{1}$	$\alpha_{2}$	$m_{1}$	$m_{2}$	$Q_{\text{max}}$ (mL)	σ	$t_{\text{delay}}$	$v_{0}$	$V_{\text{max}}$
Left Atrium	0.12	0.12	0.088	0.176	1.31	9.10	4000	0.0015	0.108	95	250
Left Ventricle	2.80	0.03	0.176	0.264	1.31	18.3	2000	0.01	0.318	130	350
Right Atrium	0.08	0.08	0.088	0.176	1.31	9.10	4000	0.0015	0.085	55	250
Right Ventricle	0.60	0.015	0.176	0.264	1.31	9.10	2000	0.01	0.318	110	350

#### 2.1.2 Interventricular septum interaction

Interventricular interaction is modeled through a septal elastance, $E_{sv}$ , which couples the pressures and volumes of the left and right ventricles (Sun et al., 1997). The left ventricular pressure, $p_{lv}$ (Equation 6), is expressed as the sum of its effective elastance contribution and the cross-talk pressure originating from the right ventricle:

$$p_{lv}=\frac{E_{sv}}{E_{sv}+e_{lv}}\cdot e_{lv}\cdot \left(v_{lv}-V_{0,lv}\right)+\frac{e_{lv}}{E_{sv}+e_{lv}}\cdot p_{rv},$$ (6)

where $E_{sv}$ denotes the septal wall elastance, $e_{lv}$ is the left ventricular elastance as defined in Equation 1, and $p_{rv}$ is the right ventricular pressure. This relation captures septal shifting during the cardiac cycle and its impact on the effective pressure within the left ventricle. The right ventricular pressure contribution and atrial septal interactions are modeled in a similar way.

#### 2.1.3 Pericardial pressure

The pericardium is modeled using an exponential pressure–volume relationship (Equation 7) to represent the mechanical constraint it imposes on cardiac filling:

$$p_{\text{pc}}\left(v\right)=p_{pc,0}+K_{pc}\cdot e^{\frac{v_{pc}-V_{pc,0}}{\varphi_{pc}}},$$ (7)

where $p_{pc,0}$ denotes the minimum pericardial pressure, $K_{pc}$ is the pericardial pressure constant, $V_{pc,0}$ represents a volume offset, $\varphi_{pc}$ is the pericardial volume constant, and $v_{pc}$ corresponds to the total pericardial volume, defined as the sum of the volumes of the cardiac chambers and the pericardial fluid volume $V_{pe}$ .

#### 2.1.4 Cardiac valves

The pressure drop across a cardiac valve is governed by Equation 8, a nonlinear inertial-resistive model (Mynard et al., 2012):

$$\Delta P=B\cdot q\cdot |q|+L\cdot \frac{dq}{dt},$$ (8)

where the resistive and inertial coefficients are defined by Equation 9:

$$B=\frac{\rho }{2A_{\text{eff}}^{2}},\;L=\frac{\rho l_{\text{eff}}}{A_{\text{eff}}},$$ (9)

where ρ = 1,060 g/ ${cm}^{3}$ is the blood density, and $l_{\mathrm{eff}}$ is the inflow length, which is set to be equal to the instantaneous opening diameter of the valve (Broomé et al., 2013). The effective valve area $A_{\mathrm{eff}}$ (Equation 10) changes dynamically based on a gating variable ζ(t) , which models the valve’s opening and closing dynamics:

$$A_{\text{eff}}\left(t\right)=\left[A_{\text{eff},\max}\left(t\right)-A_{\text{eff},\min}\left(t\right)\right]\zeta \left(t\right)+A_{\text{eff},\min}\left(t\right).$$ (10)

The dynamics of ζ(t) are governed by Equation 11:

$$\frac{d\zeta }{dt}=\left\{\begin{array}{cc} \left(1-\zeta \right)K_{\text{vo}}\Delta p,\; & \text{if }\Delta p>0\\ \zeta K_{\text{vc}}\Delta p,\; & \text{if }\Delta p<0 \end{array}\right.,$$ (11)

where $K_{vo}$ and $K_{vc}$ are the rate coefficients for valve opening and closing, respectively. Cardiac Valves parameters are reported in Table 2.

**TABLE 2:** Model parameters for cardiac valves and pericardial/septal mechanics.

Valve	$A_{\text{ann}}$	$K_{\text{vc}}$	$K_{\text{vo}}$
(a) Cardiac valve model			
Mitral Valve	5	20	40
Aortic Valve	5	20	20
Tricuspid Valve	5	30	40
Pulmonary Valve	5	30	30

$p_{pc,0}$	$K_{pc}$	$V_{pc,0}$	$\phi_{pc}$	$E_{sa}$	$E_{sv}$
(b) Pericardial and septal mechanics					
−2	1.0	320	50	12	12

#### 2.1.5 Vascular compartments

Vascular dynamics of the venous and arterial compartments are modeled using a four-element Windkessel model. The flow update at the generic $i^{th}$ vascular compartment is computed as in Equation 12:

$$\frac{dq_{\text{i}}}{dt}=\left(p_{\text{i}+1\;}-q_{\text{i }}\cdot R-p_{C}-p^{it}-\left(q_{\text{i}-1}-q_{\text{i}}\right)\cdot \Omega +\Delta p_{h}^{i}\right)/L,$$ (12)

where $q_{\text{i}-1}$ and $p_{\text{i}+1}\;i+1$ denote the intravascular blood flow and transmural pressure of the previous (i-1) and subsequent (i+1) compartment, respectively. R , Ω , and L are the resistance, viscoelastance, and inertance of the blood vessel, while $\Delta p_{h}^{i}$ is the gravitational hydrostatic contribution. The transmural pressure is a sum of the viscoelastic contribution $\left(q_{\text{i}-1}-q_{\text{i}}\right)\cdot \Omega$ , the intra-thoracic pressure pit and the pressure $p_{C}$ (Equation 13) which follows an exponential volume-pressure relation (Dahn et al., 1970):

$$p_{C}=P_{0}e^{\frac{v-V_{0}}{\varphi }},$$ (13)

where $V_{0}$ is the volume at mean pressure $P_{0}$ , while ϕ is a vessel-specific parameter which determines the non-linearity of the pressure-volume relation. The viscoelastic damping term Ω (Equation 14) depends on the vessel’s and fluid inertia:

$$\Omega =\lambda \sqrt{EL},$$ (14)

where λ=0.5 is an additional damping factor that helps in maintaining numerical stability throughout the simulation.

Geometric and vessel material properties define the resistance (Equation 15), inertia (Equation 16), and elastance (Equation 17) of each compartment:

$$R_{0}=\frac{8\cdot \eta \cdot l}{\pi \cdot r_{0}^{4}\cdot n},$$ (15)

$$I_{0}=\frac{\rho \cdot l}{\pi \cdot r_{0}^{2}\cdot n},$$ (16)

$$E_{0}=\frac{Y_{\text{inc }}\cdot h}{2\cdot \pi \cdot r_{0}^{3}\cdot l\cdot n},$$ (17)

where l , r and h are the length, radius and wall thickness, η = 0.00024 mmHg ⋅ s is the blood viscosity, $Y_{\mathrm{inc}}$ the Young’s modulus, and n is the number of parallel vessels, lumped into each compartment. The actual segmental elastances, resistances and inertances are updated in each calculation step based on the volume and radius, assuming constant vessel length and thickness.Vascular compartment - specific paraemters are reported in Table 3.

**TABLE 3:** Geometric and mechanical properties of cardiovascular compartments.

Compartment name	L, cm	R, cm	H, cm	Y, mmHg	n	P_0_, mmHg
Aortic root	2.00	1.60	0.16	2000.00	1	80
Ascending aorta	4.00	1.47	0.16	2000.00	1	80
Proximal aortic arch	2.00	1.26	0.13	2000.00	1	80
Distal aortic arch	3.90	1.19	0.12	2000.00	1	80
Descending aorta	20.00	1.10	0.10	2000.00	1	80
Peripheral arteries	30.00	0.16	0.05	2000.00	80	80
Resistance arteries	4.00	0.03	0.01	2000.00	7,000	80
Right carotid artery	20.00	0.47	0.06	2000.00	1	80
Right carotid resistance arteries	4.00	0.03	0.02	2000.00	1,200	80
Right carotid capillaries	0.02	0.00	0.00	2000.00	$2.4\times 10^{9}$	6
Right carotid vein	12.00	0.10	0.00	2000.00	1,500	4
Left carotid artery	20.00	0.41	0.06	2000.00	1	80
Left carotid resistance arteries	4.00	0.03	0.02	2000.00	1,200	80
Left carotid capillaries	0.02	0.00	0.00	2000.00	$2.4\times 10^{9}$	6
Left carotid vein	12.00	0.10	0.00	2000.00	1,500	4
Systemic capillaries	0.02	0.00	0.00	2000.00	$1.12\times 10^{10}$	6
Capacitance vessels	12.00	0.10	0.00	2000.00	7,000	4
Superior caval vein	12.00	1.40	0.15	2000.00	1	4
Inferior caval vein	20.00	1.40	0.15	2000.00	1	4
Pulmonary trunk	5.00	1.50	0.15	2000.00	1	12
Pulmonary artery	10.00	1.50	0.12	2000.00	1	12
Pulmonary resistance arteries	1.00	0.06	0.01	2000.00	2,000	12
Pulmonary capillaries	0.02	0.00	0.00	2000.00	$8\times 10^{9}$	10
Pulmonary small veins	6.00	0.08	0.00	2000.00	3,000	8
Pulmonary veins	6.00	0.70	0.10	2000.00	4	6

#### 2.1.6 Autoregulation (baroreflex)

The time-averaged aortic pressure ${\bar{p}}_{\text{ao}}$ is regulated via a baroreflex mechanism (Fois et al., 2022), modeled using sigmoidal activation functions for the sympathetic $\left(n_{s}\right)$ and parasympathetic $\left(n_{p}\right)$ neural firing rates as shown in Equation 18:

$$n_{s}\left({\bar{p}}_{\text{ao}}\right)=\frac{1}{1+{\left(\frac{{\bar{p}}_{\text{ao}}}{{\bar{p}}_{\text{ao},tg}}\right)}^{\nu }},\;n_{p}\left({\bar{p}}_{\text{ao}}\right)=\frac{1}{1+{\left(\frac{{\bar{p}}_{\text{ao}}}{{\bar{p}}_{\text{ao},tg}}\right)}^{-\nu }},$$ (18)

where ν=7 defines the steepness of the neural response, and ${\bar{p}}_{\text{ao},tg}$ is the target aortic pressure, set to 100 mmHg to match the mean arterial pressure (MAP) of the model in the supine position. These neural signals modulate a generic cardiovascular variable $y_{m}\left(t\right)$ according to Equation 19:

$$\frac{dy_{m}}{dt}=\frac{1}{\tau_{m}}\left(-y_{m}+\alpha_{m}n_{s}\left({\bar{p}}_{\text{ao}}\right)-\beta_{m}n_{p}\left({\bar{p}}_{\text{ao}}\right)+\gamma_{m}\right),$$ (19)

where $\alpha_{m}$ , $\beta_{m}$ , $\gamma_{m}$ , and $\tau_{m}$ are parameters characterizing the baroreflex dynamics specific to the regulated variable. Controlled variables include HR, systemic vascular resistance (SVR), left ventricular maximum elastance $E_{lv,max}$ , systemic venous compliance $C_{\mathrm{sys,ven}}$ , and systemic unstressed venous volume $V_{un,sys,ven}$ .

Cardiopulmonary regulation of the time-averaged right atrial pressure ${\bar{p}}_{\text{ra}}$ is implemented analogously, with a target pressure ${\bar{p}}_{\text{ra},tg}=5$ mmHg. This mechanism modulates pulmonary vascular resistance (PVR), right ventricular maximum elastance $E_{rv,max}$ , pulmonary venous compliance $C_{\mathrm{pul,ven}}$ , and pulmonary unstressed venous volume $V_{un,pul,ven}$ .

Baroreflex gains were empirically selected to match the cardiovascular blood pressure response as reported in previous work (Fois et al., 2022), which is based on *in-vivo* data (Coonan and Hope, 1983; Smith et al., 1994; Blomqvist and Stone, 1991). The relative contributions of HR, SV, CO, TPR and LVemax were also controlled via the baroreflex gain. Parameters related to the baroreflex model are reported in Table 4.

**TABLE 4:** Arterial Baroreflex and Cardiopulmonary Reflex Parameters adopted from Fois et al (2022).

$y_{m}$	$\alpha_{m}$	$\beta_{m}$	$\gamma_{m}$	$\tau_{m}$
Arterial baroreflex				
$HR/HR^{0}$	0.75	0.75	1.00	5
$E_{rv/lv,max}/E_{rv/lv,max}^{0}$	0.40	—	0.80	5
$R_{\mathrm{sys}}/R_{\mathrm{sys}}^{0}$	5.00	—	−1.50	10
$C_{\mathrm{sys,ven}}/C_{\mathrm{sys,ven}}^{0}$	−0.60	—	1.00	30
$V_{un,sys,ven}/V_{un,sys,ven}^{0}$	−0.40	—	1.20	30

$y_{cp,m}$	$\alpha_{cp,m}$	$\beta_{cp,m}$	$\gamma_{cp,m}$	$\tau_{cp,m}$
Cardiopulmonary Reflex				
$R_{\mathrm{pul}}/R_{\mathrm{pul}}^{0}$	2.50	—	−0.25	15
$C_{\mathrm{pul,ven}}/C_{\mathrm{pul,ven}}^{0}$	−0.60	—	1.30	30
$V_{un,pul,ven}/V_{un,pul,ven}^{0}$	−0.40	—	1.20	30

### 2.2 Walking-induced inertial effects of the locomotor system

Inertial effects arise from the mass of blood stored in each vascular compartment, when it is subjected to accelerations. Accelerations acting along the same direction as the vasculature will result in positive or negative blood flow. In supine positions, accelerating blood flow is driven mostly by the pumping of the heart. However, external accelerations such as gravity or body acceleration contribute to these complex blood flow dynamics. External accelerations can be conveniently modeled as local hydrodynamic pressure sources.

#### 2.2.1 Hydrodynamic pressure contribution

In walking, two accelerations act on the vascular system: gravity and body acceleration. The effect of hydrostatic pressure induced by gravity and the hydrodynamic pressure induced by body acceleration is modeled as an additional pressure head in each vascular segment:

$$\Delta p_{h}^{i}=\rho \cdot l_{i}\cdot a\cdot \cos\left(\alpha \left(t\right)\right),$$ (20)

where ρ is the blood density, $l_{i}$ is the length of the i -th segment, a is the vertical acceleration acting on the i -th segment, and α(t) is the time-dependent angle between the body axis and the gravity vector. The length $l_{i}$ of each vascular compartment is assumed to match the corresponding values reported in Table 3.

##### 2.2.1.1 Gravity contribution

The effect of gravity is implemented as a constant vertical acceleration contribution in Equation 20 as in Equation 21:

$$a=g+a_{z},$$ (21)

where $g=980\;{\text{cm/s}}^{2}$ is the gravitational acceleration and $a_{z}$ is the body acceleration.

The vertical distance from the heart to the capillaries in the right and left carotid arteries, representing the head circulation, is 24 cm. The vertical height from the heart to the systemic capillaries is given by $h_{\text{patient}}-\Delta h_{\text{head}-\text{heart}}=146\text{ cm}$ . Since the sum of the compartment lengths from the aortic root to the systemic capillaries does not add up to this height, an additional characteristic length was included in the peripheral arteries and capacitance vessels. This adjustment ensures that the hydrostatic height of the systemic arterial tree and the systemic venous tree are equal. Furthermore, the pulmonary circulation and the capillaries compartments were assumed to contribute no hydrostatic pressure, as it was considered to be oriented at an angle of 90° with respect to the gravity vector.

##### 2.2.1.2 Body acceleration contribution

An additional acceleration term complements the implementation of gravity with the body acceleration. In Equation 21 $a_{z}$ represents the dynamic vertical acceleration acting in blood vessels along the gravity vector direction.

#### 2.2.2 Intra-thoracic pressure

Intra-thoracic pressure $p^{it}$ (Equation 22) is also adjusted in the standing position, transitioning linearly from −2.5 mmHg in the supine position to −6.5 mmHg in the upright position, as a function of posture angle:

$$p^{it}\left(\alpha \right)=p_{\text{sup}}^{it}+\left(p_{\text{up}}^{it}-p_{\text{sup}}^{it}\right)\cdot \frac{\alpha }{90{}^{\circ}}$$ (22)

where $p_{\text{sup}}^{it}=-2.5\text{ mmHg}$ and $p_{\text{up}}^{it}=-6.5\text{ mmHg}$ .

### 2.3 Experimental human-subject data

Highly controlled experimental human-subject data served as qualitative validation for the walking induced-inertial effects as simulated in the cardiovascular model.

Two subjects were included in the study (P1: male, 31 years old, height 177 cm, weight 63 kg, P2: male, 26 years old, height 168 cm, weight 65 kg). Subjects were chosen from a larger cohort 1) to have a similar height as our cardiovascular closed-loop model implementation and 2) to depict a distinctly different degree of hemodynamic response to synchronized exercise. Ethical approval was obtained from the Swedish Ethical Review Authority (2023-00426-01), and the participant provided written informed consent. Continuous finger arterial pressure was obtained via Finapres (Finapres Medical Systems B.V., Netherlands), with height correction unit enabled and reconstruction of the brachial arterial pressure signal. Simultaneously, electrocardiogram (ECG) was acquired from a chest sensor, equipped with inertial measurement units (IMU) and pressure insoles were used for step timing detection (Cometa Srl., Italy). All data were wirelessly transmitted for acquisition at 2 kHz.

To guide heart-paced walking, the subjects wore a chest strap (Pulson, USA) equipped with ECG and IMU sensors. The device played auditory tones timed to coincide with the R wave (systole) or at 45% of the R-R interval (diastole). The experimental protocol consisted of a 15-min treadmill walking. The initial 3 min served as a warm-up, followed by two 3-min bouts each of systolic and diastolic stepping, totaling 12 min of heart-paced walking.

### 2.4 Protocols

Three sets of protocols were run in this study. First, a tilt test involving a head-up tilt of 90° from lying down to standing was performed to validate the implementation of baroreflex and gravity into the cardiovascular model. Second, a synthetic walking waveform protocol at controlled HR and SRs. In this setup, the inertial effects on hemodynamics were studied by controlling both HR and SR. The walking pattern was reconstructed as a time-averaged cyclic waveform based on subject measurements and applied as a periodic acceleration input at the specified SR frequency. Third, a human-subject walking protocol to capture a real-world scenario of heart-paced walking. We compared hemodynamic features arising from the effects of heart-paced walking in the simulated descending aortic pressure with finger-measured brachial blood pressure. As input, the simulation used real-world HR and chest acceleration data from the subjects.

In both walking protocols, the baroreflex control acting on the HR was deactivated. This was done either because the HR was imposed as fixed (in synthetic walking) or was measured from the human-subject. All other baroreflex controls remained active.

#### 2.4.1 Tilt test

We conducted a tilt test simulation to validate our integrated baroreflex and gravitational model against published physiological data (Fois et al., 2022). For this purpose, mean values of main pressures, including MAP, systolic arterial pressure (SAP), diastolic arteral pressure (DAP) and central venous pressure (CVP) were compared against mean values from experimental literature and computational data from Fois et al. (2022). Percentage change in HR, SV, CO, total peripheral resistance (TPR), and $E_{lv,max}$ was also compared.

Postural transition was modeled as a 90° head-up tilt from the supine position, following the methodology described in Heldt (2004).

The postural angle α(t) (Equation 23) was modeled using a smooth cosine transition to emulate the physiological tilt maneuver:

$$\alpha \left(t\right)=\left\{\begin{array}{cc} \alpha^{\text{sup}},\; & t<t_{\text{tilt},0}\\ \alpha^{\text{sup}}+\frac{\alpha^{\text{up}}-\alpha^{\text{sup}}}{2}\left(1-\cos\left(\pi \frac{t-t_{\text{tilt},0}}{\Delta t_{\text{tilt}}}\right)\right),\; & t_{\text{tilt},0}\leq t\leq t_{\text{tilt},0}+\Delta t_{\text{tilt}}\\ \alpha^{\text{up}},\; & t>t_{\text{tilt},0}+\Delta t_{\text{tilt}} \end{array}\right.$$ (23)

where $\alpha^{\text{sup}}=0{}^{\circ}$ is the supine angle, $\alpha^{\text{up}}=90{}^{\circ}$ is the standing angle, $t_{\text{tilt},0}$ is the tilt initiation time, and $\Delta t_{\text{tilt}}$ is the duration of the postural change.

#### 2.4.2 Synthetic walking

Synthetic acceleration signals were computed by repeating one vertical acceleration cycle and were provided as input to the model. In all simulations, the HR was fixed at 70 bpm. The pressure in the descending aorta was selected for this analysis.

Initially, the SR was set equal to the HR, with five distinct phase shifts to the cardiac cycle. The first phase shift occurred when the peak acceleration coincided with mitral valve closure, the third phase shift corresponded to the alignment of peak acceleration with ventricular filling, marked by maximal mitral valve opening, and the last phase shift when the peak acceleration occurred just before the mitral valve closure of the subsequent heart beat. The second and fourth shifts occurred during the transitions between systole and diastole and diastole and systole.

Subsequently, the SR was increased beyond HR by 10%, 20%, and 30%. The difference in frequency, naturally iterates through the different phase shifts of the two physiological systems. Fast Fourier Transform (FFT) was performed to compare the frequency content of the pressure signals between the standing and walking conditions. FFT resolution was set as 0.02 Hz.

#### 2.4.3 Human-subject walking

The experimentally measured chest acceleration and HR data from the subjects were used as inputs to the computational model. The simulated pressure in the descending aorta was then compared to the brachial arterial pressure.

To determine the phase synchronization between step events and the cardiac cycle, the phase of each step was calculated as the percentage of the RR interval at which the heel strike occurred. Heel strike timing was identified from insole data as the point at which average pressure reached a certain threshold, calculated with Cometa proprietary software. Cardiac contraction was defined as the R-peak in the ECG for experimental data and as the mitral valve closure in the simulated data. A phase of 0% corresponds to a step occurring simultaneously with cardiac contraction, while 100% indicates a step coinciding with the subsequent contraction.

Pulse waveforms were segmented to each phase, starting from heart contraction to 35%RR after the subsequent contraction. The mean waveform for each phase was then computed for further analysis. The experimental waveform was shifted 670 samples for alignment purposes.

### 2.5 Statistical and waveform analysis

A two-sample Kolmogorov-Smirnov (K-S) was performed to compare beat-wise experimental and simulated waveforms with and without hydrodynamic pressure contribution in the cardiovascular model. The K-S test was chosen, as it emphasizes pressure level distribution over one cardiac cycle and de-emphasizes waveform timing when compared to correlation analyses. Each hemodynamic time-series was segmented into waveforms from one pressure onset to the subsequent one and then concatenated. Pressure onset was identified as the projection on the signal of the intersection between a horizontal line passing through the minimum before the start of the systolic upstroke, and the tangent to the point of maximum first derivative (Chiu et al., 1991). The K-S test statistic served as a similarity index (K-stat), quantifying the maximum absolute difference between the empirical cumulative distribution functions of the experimental and simulated hemodynamic waveform for each subject and condition.

## 3 Results

### 3.1 Gravity and baroreflex autoregulation

**FIGURE 2:**
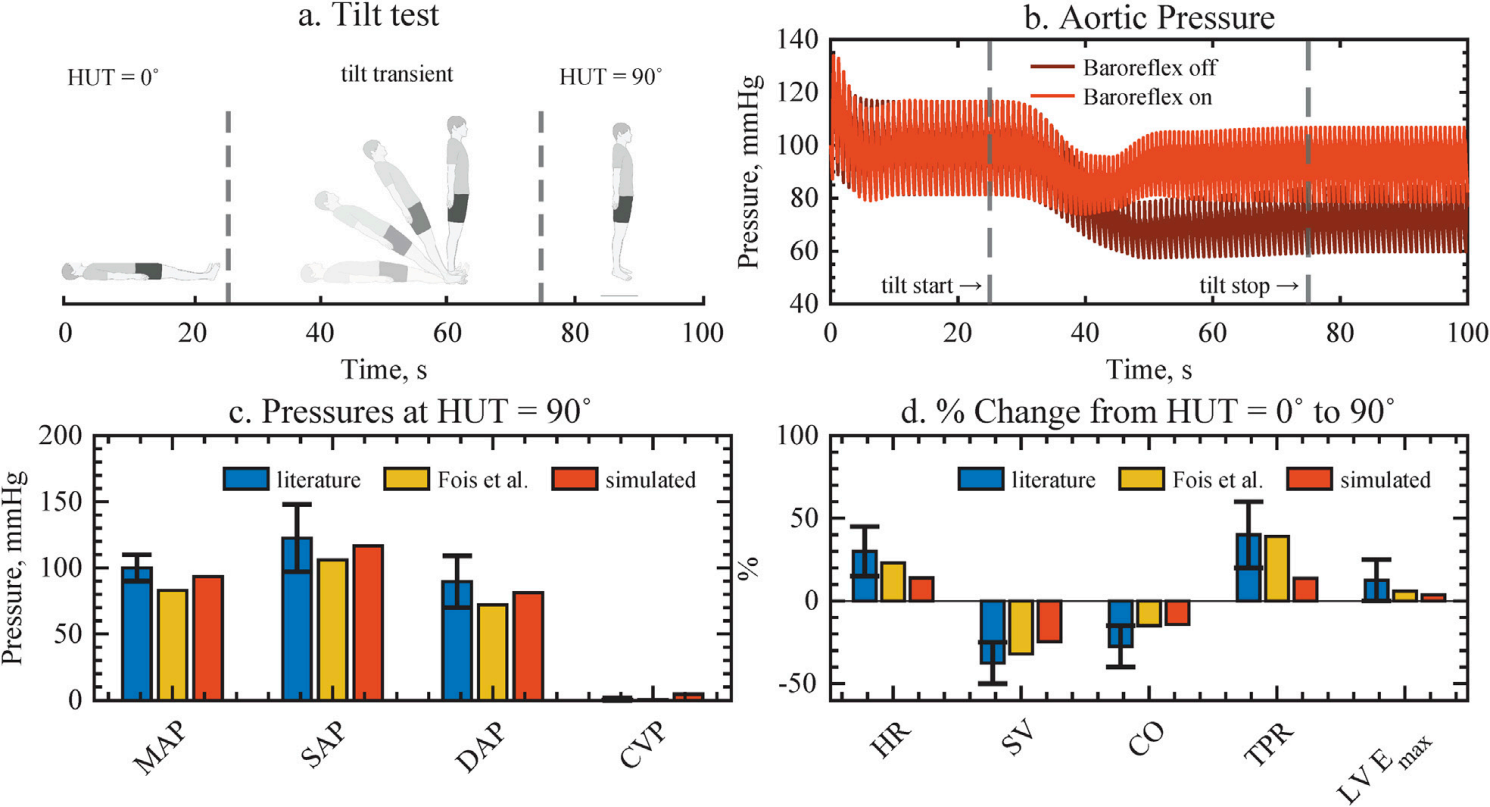
Validation of gravity and baroreflex autoregulation against assembled experimental data from literature and previous computational model implementations, resulting in good agreement for all cardiovascular variables. **(a)** Head-up tilt protocol. **(b)** The aortic pressure response with and without baroreflex, depicting how pressure is restored when the baroriflex is active. **(c,d)** Pressure values and physiological changes upon tilting, compared to literature and previous computational model. MAP, mean arterial pressure; SAP, systolic arterial pressure; DAP, diastolic arterial pressure; CVP, central venous pressure; HR, heart rate; SV, stroke volume; CO, cardiac output; TPR, total peripheral resistance; LVEmax, left ventricular contractility.

The implementation of gravity and baroreflex autoregulation was validated with pre-published experimental and simulation data from literature. A head-up tilt served as the validation experiment. Figure 2B illustrates the physiological aortic pressure response of the computational model to standing up with the baroreflex on versus baroreflex off. Upon standing up, blood pools in the lower extremities, leading to a reduction in venous return, SV, and CO. As a result, aortic pressure drops. This gravitational effect on hemodynamics is noticeable about 10 s after the start of the tilt (see Figure 2B), a realistic scenario given previously published data. We conclude that the hydrodynamic pressure source is correctly implemented.

As a physiological response, the drop in aortic pressure is registered by the stretch-sensitive barorecepters, which trigger compensatory autoregulatory mechanisms. Autoregulation increases HR, total vascular resistance, and cardiac contractility in order to maintain arterial pressures. Consequently, the baroreflex control of our computational model increases all arterial pressure similarly to previously reported levels when the baroreflex is on (see Figure 2C). In contrast, in the absence of baroreflex control, there is a noticeable and sustained decrease in pressure. Additionally, a physiological decrease in pulsatility can be noticed in both cases. Furthermore, the global hemodynamic changes in HR, SV, CO, total peripheral resistance, and left ventricular contractility to achieve arterial pressure control agree with aggregated experimental data and previous computational model results (Fois et al., 2022). While the relative magnitude of the changes is smaller in our model compared to literature, it accurately reproduces the dynamics of the hemodynamic transient of a head up tilt.

### 3.2 Walking-induced hemodynamics in steady state

#### 3.2.1 Equivalence of SR and HR

**FIGURE 3:**
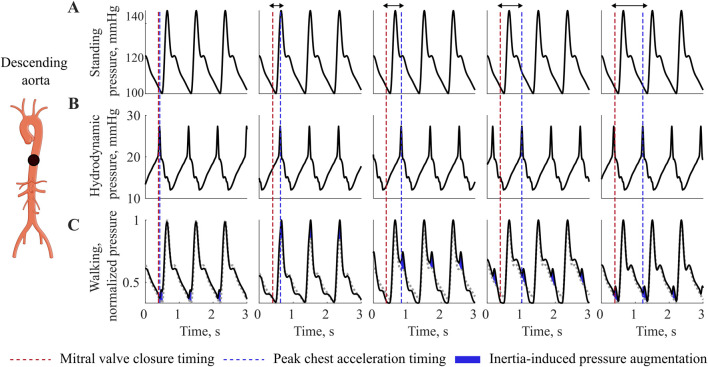
Interaction at equivalence of HR and SR. Pressure variations during walking at different phase relationship between heart contraction and maximal acceleration, showing pressure augmentation during walking compared to resting. **(A)** Standing pressure, **(B)** Walking hydrodynamic pressure, **(C)** Walking pressure. Red dotted line depicts the mitral valve closure timing, while the blu dotted line depicts the timing of chest acceleration peak related to the walking movement. Blue, inertia-induces pressure augmentation.

At rate-equivalence, inertial effects associated with walking are evident in the normalized pressure waveforms of the descending aorta, as illustrated in Figure 3. The Figure shows increasing phase shifts (arrows) between the heart contraction (red) and the maximum body acceleration (blue). Compared to the baseline waveform while standing (Figure 3A), walking introduces an additional hydrodynamic pressure component (Figure 3B), resembling the waveform shape of the chest acceleration. As the acceleration peak shifts with respect to the cardiac contraction, the resulting inertia-induced pressure augmentation also shifts towards the diastolic part of the waveform to the right. Depending on the phase shift between cardiovascular and locomotor activity, and thus on the timing of this interaction, the increase is observed either in systolic or diastolic pressure, (Figure 3C).

#### 3.2.2 SR higher then HR

**FIGURE 4:**
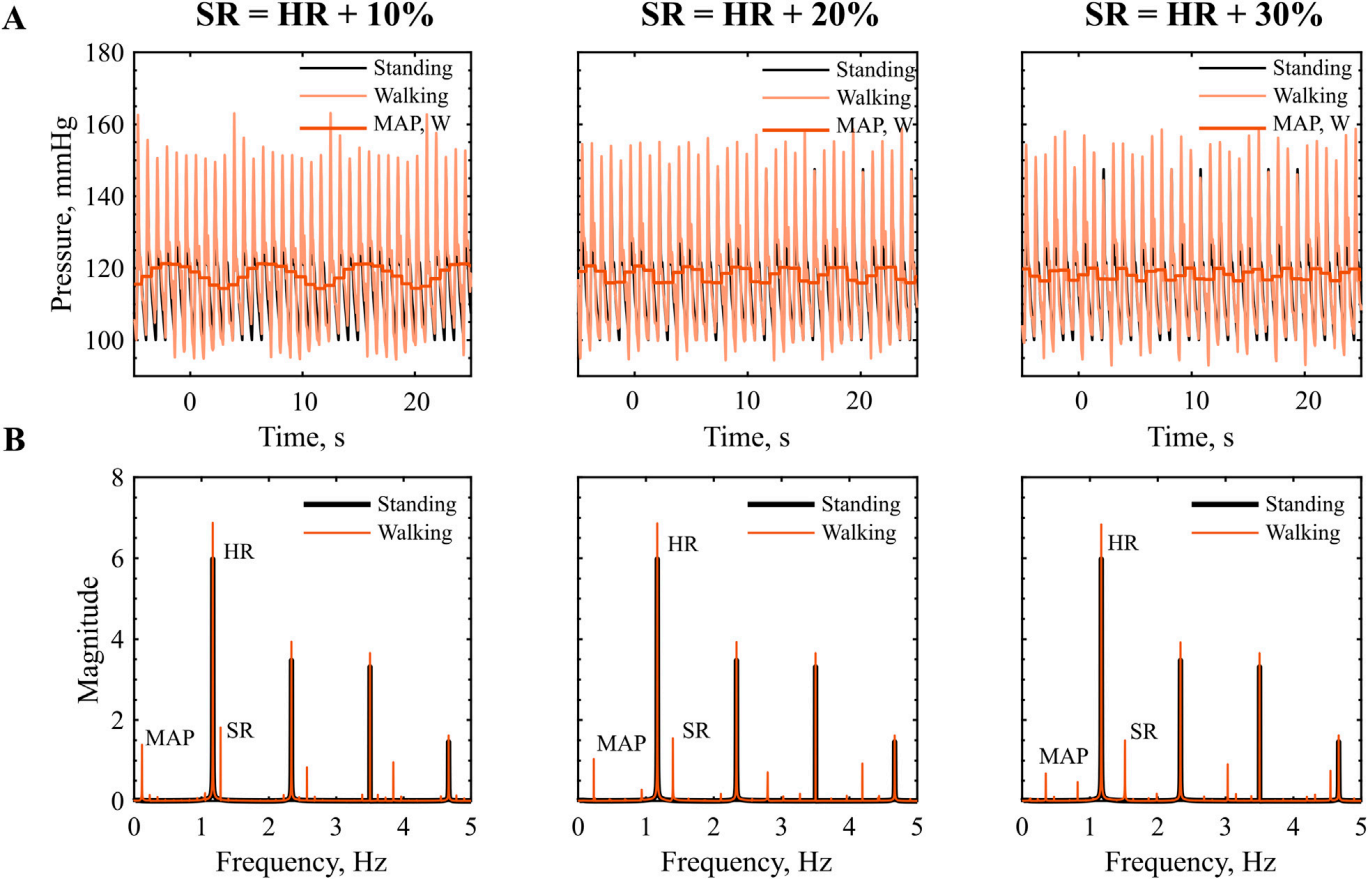
Interaction at SR higher then HR: **(A)** Time series of pressure in the descending aorta during walking, red, vs. standing, black. **(B)** Frequency amplitude spectra, prominent peaks indicate the dominant frequency components in the signal. HR = 70 bpm, SR = 77, 84, 93 steps/min.

When the SR exceeds the HR, the phase is no longer constant and we expect higher order system interactions between two systems of similar frequency. In the time-series signal, we observe a low frequency component in the pressure waveform, characterized by a frequency lower than both HR and SR, Figure 4A. The MAP also reflects this interaction, exhibiting a sinusoidal pattern, with low frequency.

The frequency spectrum in Figure 4B reveals distinct peaks at the SR the HR and their higher order harmonics. As presented in Table 5, we can link most frequency peaks to the imposed HR and SR. The dominant low frequency is associated with the lower frequency observed in the MAP. The value is equal to the difference between SR and HR across all conditions tested, which suggests that the signal results from a ‘beating’ interaction between the HR and SR. However, the addition of the two frequencies does not result in a frequency peak. Additional low-frequency components emerge in the range between the ‘beating’ frequency and the HR. The low magnitude of these components (f < 0.1 mmHg) suggests that they might be associated with harmonics of the beating frequency or could result from numerical aliasing effects.

**TABLE 5:** Frequency content of the pressure waveform in the descending aorta in standing and different walking frequency. HR = 70 bpm, SR = 77, 84, 93 steps/min.

	Frequency (Hz)			
	Standing	HR+10%	HR+20%	HR+30%
SR-HR		0.117	0.231	0.349
		*0.231*		
		*0.347*		
		*1.053*	*0.937*	*0.819*
HR	**1.167**	**1.167**	**1.167**	**1.167**
SR		**1.283**	**1.397**	1.517
2*HR	**2.335**	**2.335**	**2.335**	**2.335**

Bold, magnitude >1.5 mmHg, regular magnitude >0.5 and <1.5 mmHg, italics magnitude <0.5 mmHg.

### 3.3 Experimental validation with heart-paced walking

**FIGURE 5:**
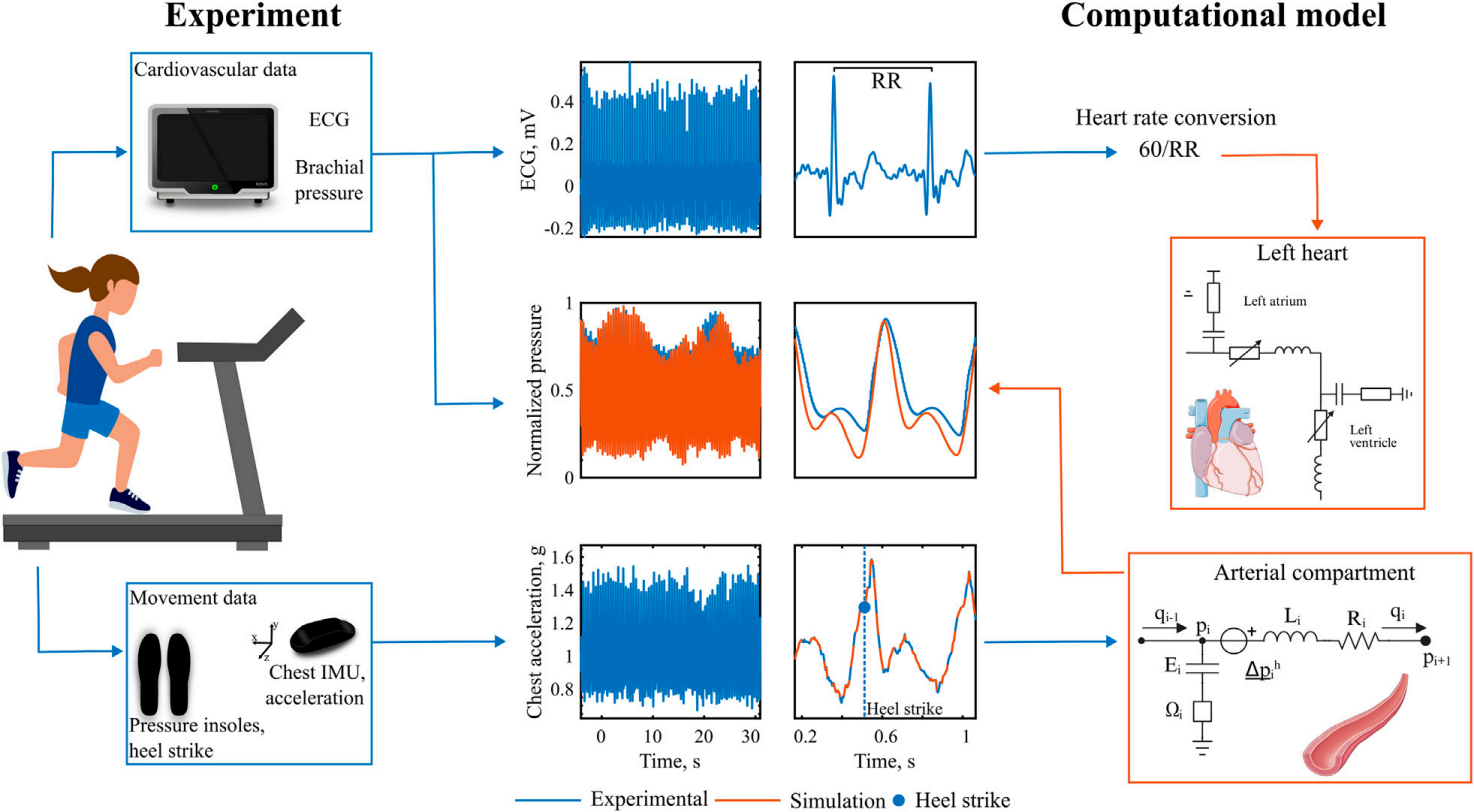
Experimental validation with heart-paced walking. Data recorded during heart-paced treadmill walking. The experimental chest acceleration and heart rate data are fed into the computational model, which output the simulated pressure. Experimental recorded brachial pressure is then compared with the simulated pressures.

The experimental validation methodology and collected data are presented in Figure 5. The model receives time series inputs of HR, derived from the ECG, and vertical chest acceleration, and generates an estimate of arterial pressure. This simulated pressure is then compared against experimentally measured brachial pressure. As illustrated in the 30-s snapshots in Figure 5, the simulated pressure accurately captures the amplitude modulations associated with walking activity, exhibiting consistent narrowing and elevation of both systolic and diastolic pressure peaks. Heel strike events, identified from pressure insole data, are used to determine the precise timing of each step and to characterize the phase relationship between the cardiac and gait cycles.

**FIGURE 6:**
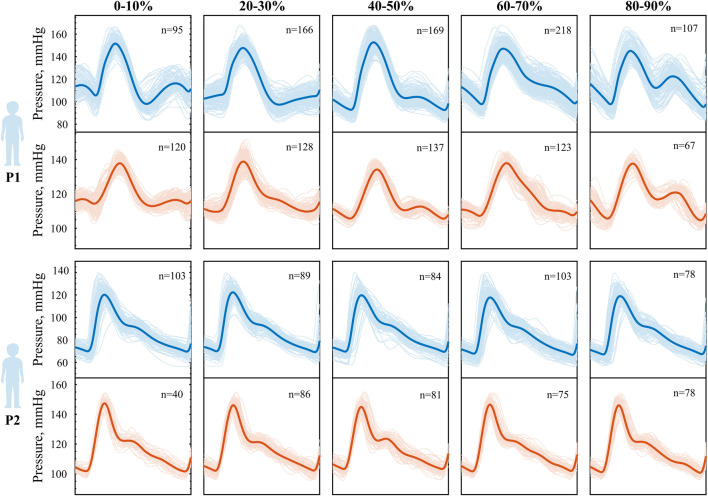
Phase-modulation during heart-paced walking in **P1** above and **P2** below. Averaged pressure waveform for different phase relationship between step and cardiac contraction, experimental (blue) vs. simulated (orange). Phase 0% indicates heel strike coinciding with cardiac contraction, while phase 100% indicated heel strike coinciding with the subsequent cardiac contraction.

A more detailed analysis of pressure amplitude modulations induced by inertial effects at varying phase shifts reveals distinct patterns in the pressure waveform. These patterns become evident when the data are grouped according to the phase relationship between cardiac contraction and peak body acceleration, as shown in Figure 6. We observe two distinct pressure responses to synchronized walking in the two subjects. In P1, during early phases (0%–10%), a small pressure elevation appears just before the onset of the main systolic upstroke. As the phase between the two signals increases, the timing of the pressure augmentation shifts within the cardiac cycle. In mid-phase intervals (40%–50%), it manifests as an augmented systolic peak, while in later phases (60%–90%), it contributes to an elevated diastolic peak pressure. Notably, in the final phase (90%–100%), the pressure waveform adopts a distinct bi-phasic shape, similar to the pattern observed in the initial phases (0%–10%). In addition to the variability in waveform shape, we further observe a variable pressure pulsatility depending on the phase. In P2, instead, the waveform morphology does not show visible changes across all phases. However, the small variability present is captured well in the simulated waveform.

The waveforms were constructed as an average over a significant number of beats (40 < n > 218). Pressure waveform variability across these beats is observed to a similar extent across all phases. The systolic upstroke in the pressure waveform exhibits the least variance, indicating highest consistency in its timing and shape. In contrast, the variability is greater across augmentations in the diastolic part of the cardiac cycle, after the systolic peak. The variability is particularly high in phases 60%–90%, where even slight differences in the stepping timing (within 10%RR) lead to noticeable changes in the diastolic waveform morphology. We associate this to the generally lower pressure levels in diastole.

**FIGURE 7:**
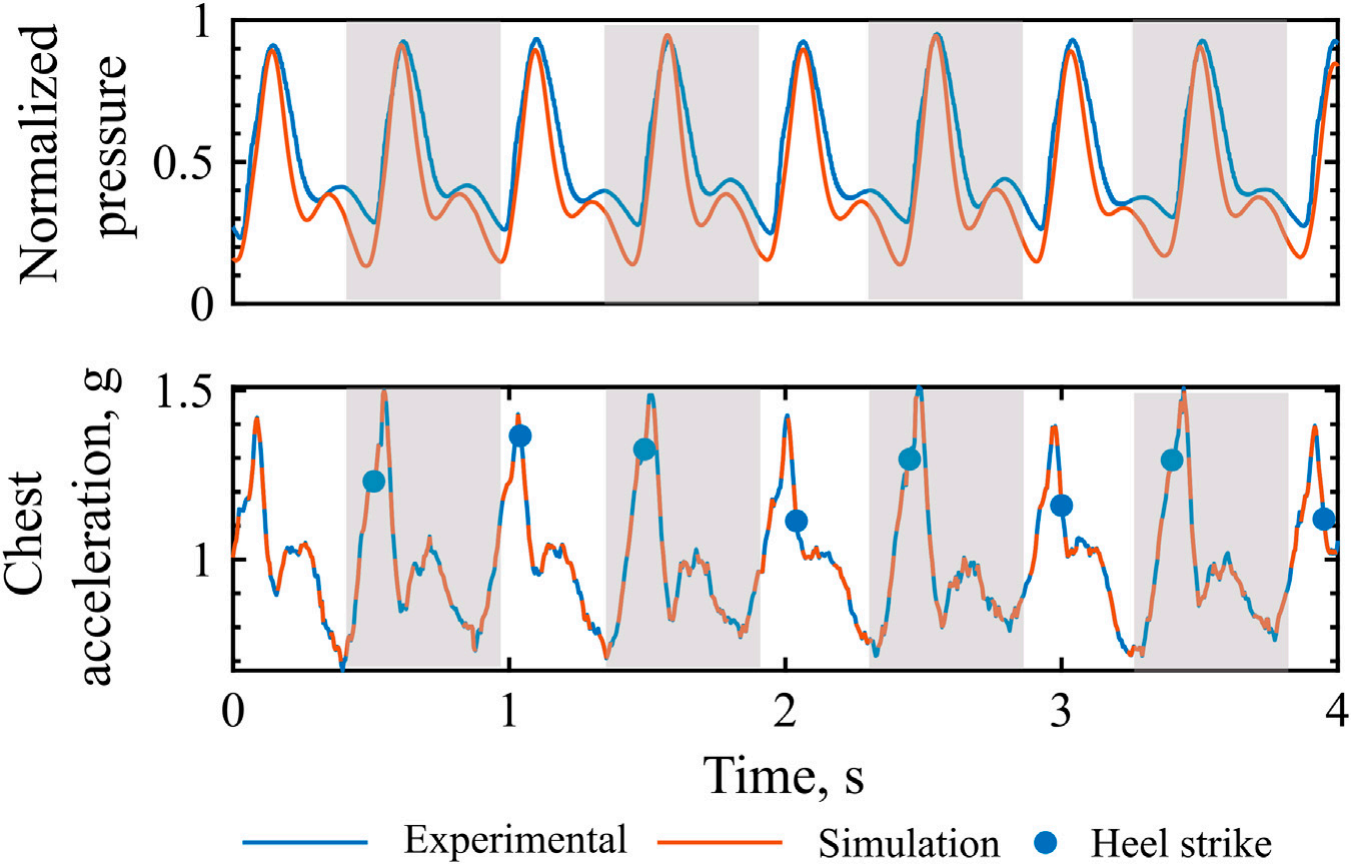
Extent of pressure augmentation: Experimental vs. simulated pressure waveform and chest acceleration. Grey, left leg step.

The computational model of walking accurately reproduces the extent of pressure amplitude modulation induced by variable amplitude of the inertial effects, as noticeable in Figure 7, P1. In the reported example, peak body accelerations are slightly different between right and left-sided stepping, such that the amplitude of the walking-induced body acceleration influences the diastolic peak amplitude. It can be qualitatively observed that both the experimental and simulated pressure waveform closely follow this alternating pattern: a lower systolic peak when peak body acceleration is low, and a reduced dicrotic notch together with an elevated diastolic peak when the acceleration trough, corresponding to the toe-off phase of the walking cycle, becomes more pronounced. The cyclic alternation of this behavior could be due to different push-off intensity of the two legs.

#### 3.3.1 Waveform morphology

**FIGURE 8:**
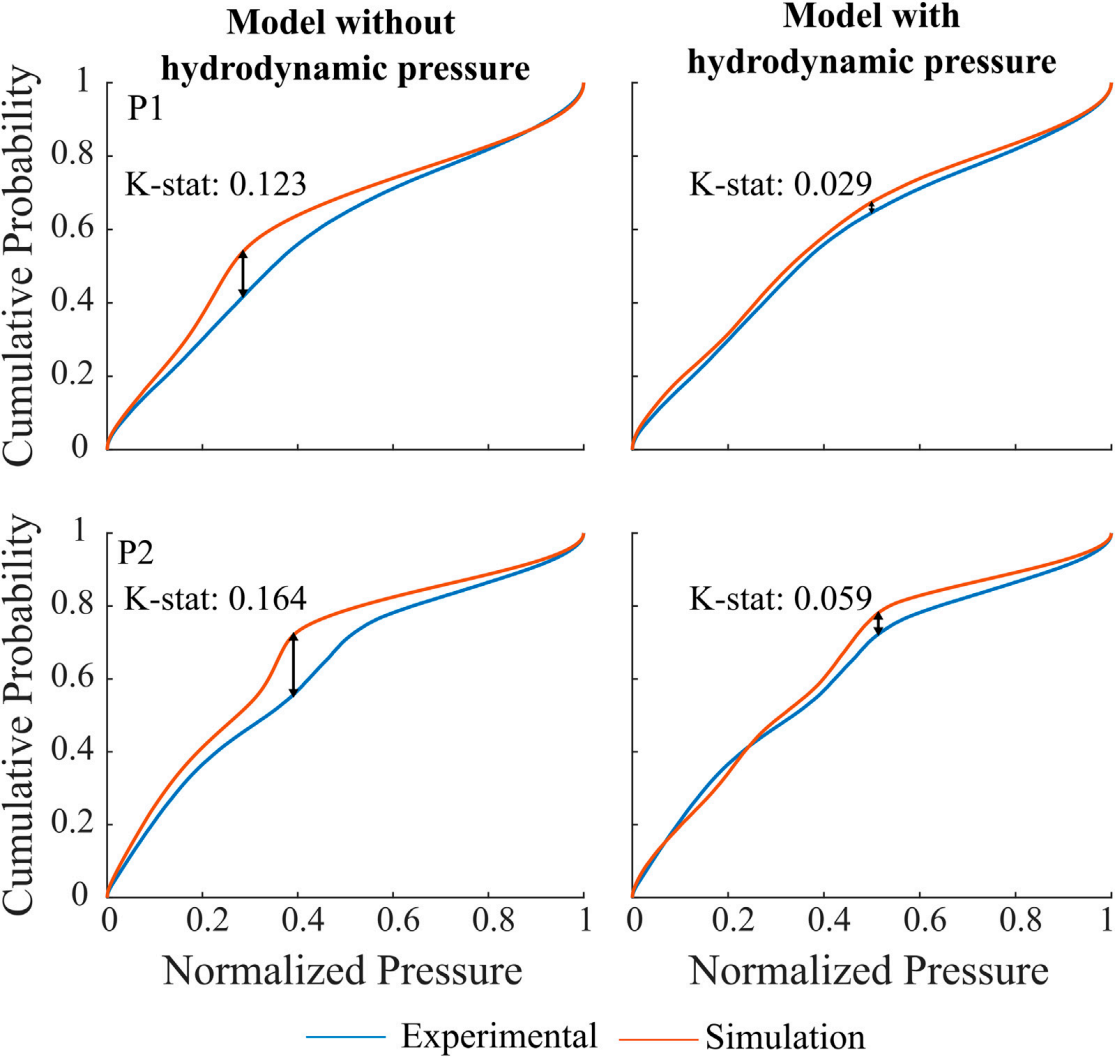
Cumulative distribution function and K-stat for P1 and P2 when hydrodynamic pressure is not implemented, left vs. when hydrodynamic pressure is implemented in the model, right. The arrows represent the maximum difference between the experimental and simulated time series.

A total of 1.120 waveforms for P1 and 750 for P2 were included in this analysis. The similarity index and cumulative distribution function are shown in Figure 8 for both participants. The morphology results indicate an increase in similarity index when hydrodynamic pressure is incorporated in the model for both subjects (K-stat 0.123 vs. 0.029, P1 and 0.164 vs. 0.059, P2). The distributions are closely aligned for the two extremes 0 and 1, representing pressure onset and systolic peaks. Larger difference is instead observed for pressure values between 0.4 and 0.6, most likely to occur at the diastolic phase of cardiac cycle. It is further noted that all pairwise comparisons between simulation and experiment still indicate that the distributions remain significantly different (p < 0.05).

## 4 Discussion

We have demonstrated the ability to computationally model walking-induced inertial effects on the blood pressure waveforms of the cardiovascular system. The motion-induced inertial component of the blood re-acting to body acceleration, alters waveform morphology from the commonly known resting blood pressure waveforms.

Our model reproduces pressure waveform morphology in standing and during walking, which was validated with illustrative high quality experimental time-series data in two subjects. The implementation of gravity and baroreflex autoregulation has been validated against literature. Although the autoregulation response is slightly smaller in our model, possibly due to different gain of the feedback loop, it accurately captures all of the known cardiovascular responses in a physiological way. More importantly, comparison of pressure waveforms during walking between model and experimental data exhibited high similarity in both morphology, with an emphasis of timing of the augmentation peak and relative amplitude. Furthermore, the extent of pressure augmentation was seen to be related with peak body acceleration amplitude in both experiment and simulation. Hydrodynamic pressure contribution improves the degree of similarity of the resulting waveform morphology. We conclude that introducing contributions of body acceleration as an additional dynamic component of the pressure source in the vascular compartments, seems a valid way to capture walking induced inertial effects.

Locomotor and cardiovascular function are linked via a variety of mechanism including the venous muscle pump, body acceleration induced inertial effects as well as potentially altered peripheral wave reflection timing during rhythmic muscle contraction. Our results indicate that inertia-induced effects play a major role in this interaction. Previous studies reported observations of ‘beating’ of the arterial pressure waveform during running but not during cycling (Palatini et al., 1989a; Palatini et al., 1989b), which was unrelated with respiration. Their results further support the hypothesis that the effect is driven by gravity-induced inertial forces. Interestingly, their study quantified the hydrodynamic pressure component using a saline-filled container, reporting values ranging from approximately 10–65 mmHg. In our simulations, the hydrodynamic pressure contribution was estimated to be in the range from 15 to 25 mmHg, consistent with the values observed experimentally. During running, we anticipate more pronounced body accelerations, which would lead to greater inertial forces and correspondingly higher hydrodynamic pressure components.

In a separate study, researchers (O’Rourke et al., 1993) recorded pressure variations induced by running during both systolic and diastolic phases and compared them to pressure waveforms generated by an intra-aortic balloon pump. They found that running with diastolic phase acts as a counterpulsation, closely resembling the effect of the intraortic balloon pump, timed to reduce systolic and increase diastolic pressure. The characteristic shapes observed in O’Rourke recordings closely resemble those found in our study. Specifically, higher pulse pressure have monophasic shape, while lower pulse pressure biphasic.

Our study was designed with a high degree of synchronization between movement and heart rate. This allowed us to stratify the pressure waveforms into a wide range of phases and observe subtle, beat-to-beat hemodynamic effects. The model was able to follow these phase-related pressure changes and showed good agreement with experimental patterns. However, despite the qualitative similarity in waveform shape, the magnitude of changes differed between experimental and simulated data. This discrepancy is likely due to differences in the lengths and properties of cardiovascular compartments that have not been accounted for. Additionally, other mechanisms, such as variation in skeletal muscle activation, not currently represented in the model may also play a role in pressure modulation. Longer time-scale regulatory mechanisms, such as metabolic perturbations and hormonal control, are also not implemented in the model. As a result, slower compensatory adaptations are not captured.

Our simulation’s results with synthetic acceleration allowed for detailed study of possible physiological interactions between the cardiovascular and locomotion system dynamics. Low frequency ‘beating’ effects were identifiable in the resulting spectrum when the SR exceeded the HR. Consistent with previous findings by Palatini et al. (1989b), the beating frequency corresponds to the difference between HR and SR. In their experiments, MAP remained stable; in contrast, our simulations revealed a reduction in MAP with an increased difference between SR and HR, resulting in lower-frequency oscillations in the pressure waveform. These results are considered exploratory and will require further experimental validation. The physiological implications of such lower frequency behavior remain an open topic for investigation.

Limitations of the study include a small number of subjects against which the model was validated. We mitigate some of this limitation, by closely controlling the experimental condition, allowing for a beat-to-beat analysis and by analysing over a significant number of beats and steps. The observations made in this study regarding pulse waveform characteristics require further validation through additional quantitative measurements, for example, analyzing the timing and magnitude of the pressure augmentation in relation to specific features of the acceleration signal in a representative population. The inertial effects identified are primarily expressed as pressure augmentation or reduction within the major aortic arteries. However, it is likely that walking also influences other key cardiovascular parameters, including preload, cardiac filling and afterload. The potential physiological effects of optimizing the phase relationship between walking and cardiac function remain an open question that deserves further investigation. This study actively refrains from drawing too many conclusions about the closed-loop cardiovascular effects of walking on the cardiovascular system, as the limited amount of subjects would render this highly speculative. Additionally, in real-world walking, CLC emerges from bidirectional interactions, where both cardiac and locomotor rhythms influence one another in real time. As we are not modeling bidirectional interactions, the findings derived from heart-paced walking may not fully reflect the dynamics of cardio-muscular coordination in free movement.

## 5 Conclusion

In conclusion, we present here a closed-loop cardiovascular model, which allows the study of dynamic pressure phenomena induced by bodily acceleration in hemodynamic waveforms.

## Data Availability

The raw data supporting the conclusions of this article will be made available by the authors, upon reasonable request.
